# Metabolic Syndrome Exacerbates the Recognition Memory Impairment and Oxidative-Inflammatory Response in Rats with an Intrahippocampal Injection of Amyloid Beta 1–42

**DOI:** 10.1155/2018/1358057

**Published:** 2018-08-01

**Authors:** Alfonso Diaz, Claudia Escobedo, Samuel Treviño, Raúl Chávez, Gustavo Lopez-Lopez, Carolina Moran, Jorge Guevara, Berenice Venegas, Guadalupe Muñoz-Arenas

**Affiliations:** ^1^Facultad de Ciencias Químicas, Benemérita Universidad Autónoma de Puebla, Puebla, PUE, Mexico; ^2^Departamento de Bioquímica, Facultad de Medicina, Universidad Nacional Autónoma de México, Ciudad de México, Mexico; ^3^Departamento de Biología y Toxicología de la Reproducción, Instituto de Ciencias, Benemérita Universidad Autónoma de Puebla, Puebla, PUE, Mexico; ^4^Facultad de Ciencias Biológicas, Benemérita Universidad Autónoma de Puebla, Puebla, PUE, Mexico

## Abstract

An important worldwide health problem as the result of current lifestyle is metabolic syndrome (MS). It has been shown that MS induced by a high-calorie diet (HCD) in rats produces cognitive deterioration in the novel object recognition test (NORt) and decreases synaptic connections and dendritic order in the hippocampus and temporal cortex. However, it is unknown whether MS induced by an HCD participates in the cognitive process observed with the injection of A*β*_1–42_ into the hippocampus of rats as a model of Alzheimer disease (AD). The induction of MS in rats produces a deterioration in NORt; however, rats with MS injected with A*β*_1–42_ show a major deterioration in the cognitive process. This event could be explained by the increment in the oxidative stress in both cases studied (MS and A*β*_1–42_): together, the hippocampus and temporal cortex produce an enhancer effect. In the same way, we observed an increment in interleukin-1*β*, TNF-*α*, and GFAP, indicative of exacerbated inflammatory processes by the combination of MS and A*β*_1–42_. We can conclude that MS might play a key role in the apparition and development of cognitive disorders, including AD. We propose that metabolic theory is important to explain the apparition of cognitive diseases.

## 1. Introduction

Metabolic disorders such as atherosclerosis, cardiovascular disease, type 2 diabetes mellitus (T2DM), and metabolic syndrome (MS) are the result of current population lifestyle [1]; these are associated with body fat accumulation, hypertension, dyslipidemia, and hyperglycemia. MS is considered the precursor of T2DM and is the result of excessive consumption of saturated fatty acids and refined sugars [2]. MS is characterized by obesity, insulin resistance, a high concentration of triglycerides, and cholesterol [3] that at central level entail endoplasmic reticulum stress in neurons [4] and cognitive disorders [5].

On the other hand, it has been shown that inflammation (peripherical or central) is associated with metabolic diseases [6]. Inflammation can induce the release of the cytokine tumor necrosis factor-alpha (TNF-*α*) [7], which produces insulin resistance by the inhibition of the insulin receptor tyrosine kinase activity [8]. Likewise, TNF-*α* increases the secretion of interleukin- (IL-) 1*β* and IL-6, which decreases adiponectin secretion [9]. This inhibition results in a decrease in fat oxidation and an increase in inflammatory molecules [10]. Argente-Arizón et al. [11] demonstrated that obesity caused by the consumption of a high-fat diet induced an increase in insulin resistance and hypothalamus inflammation. In this sense, it is known that at the central level, microglial activation decreases neuronal plasticity and synapse formation [12], as well as inflammation, and can increase tau phosphorylation [13] and amyloid beta (A*β*) formation.

MS also produces oxidative stress (OS), which is defined as an imbalance between the production and reduction of free radicals from oxygen species in biological systems. The disturbances in the normal redox status of cells cause an increment in reactive oxygen species (ROS) [14]. Interestingly, MS development in rats fed with fructose [15] or a high-calorie diet (HCD) [16] produces an increment in ROS. Therefore, OS could be the link between MS and cognitive disorders [13].

Alzheimer disease (AD) is the most common cause of dementia and cognitive disorders and is one of the most important causes of morbidity and mortality among the aging population [17]. It is characterized by extracellular senile plaques of A*β* peptides and intracellular neurofibrillary tangles of tau protein [18, 19]. Diverse factors have been related to AD development, but OS and inflammation invariably are involved [13, 14].

It has been shown that MS can participate in the development of AD [5, 20]. The evidence strongly suggests that factors such as obesity, hyperglycemia, insulin resistance, adiposity, and hypertension are related to the appearance of AD [21]. Mice fed with a high-fat diet for a long time develop obesity, insulin resistance, and elevated levels of A*β* peptides in the brain [5, 22]. Recently, it has been shown that the employment of a HCD in rats produces MS and a deterioration of the cognitive process, which could be the result of the loss of synaptic connections [23]. There is therefore increasing support for thinking that MS and its complications are key factors in the appearance of AD.

Therefore, we propose that inflammatory events and OS that result from MS are crucial factors in accelerating cognitive deficiency in rats administered with A*β*_1–42_ into the hippocampus.

## 2. Methodology

### 2.1. Animals

Male Wistar rats (70–100 g) obtained from the “Claude Bernard” vivarium of the University of Puebla were used for this study. The animals were housed in a controlled temperature and humidity environment, with light-dark cycles of 12 : 12 hours for 15 days with free access to food and water. All treatment methods used in this study were performed according to the guide for the care and use of laboratory animals (NOM-062-ZOO-1999), in addition to strictly following the guidelines for the use of animals in neuroscience research.

### 2.2. Metabolic Syndrome Induction

The animals were divided into two groups: normal-calorie diet (NCD) and HCD (*n* = 20/group). Both diets were administered for 90 days “ad libitum.” The hypercaloric diet (patent: MX/E/2013/047377) contains 71.4% carbohydrates. The percentage composition corresponds to glucose (80%) and fructose (20%). The fat content corresponds to 5.8% divided mainly between monounsaturated and saturated fats, as well as polyunsaturated fatty acids, omega 3 and 6, and the protein content was provided by 7.3% ovalbumin (Table 1). The diet did not contain a raw fiber extract. LabDiet 5010 (laboratory rodent diet) was used as the NCD; the composition can be consulted on the manufacturer's website. The HCD provides the nutrimental requirements established for laboratory rats by the National Academy of Sciences [16, 23].

### 2.3. Zoometric Parameters

The weight and height of the rats were monitored weekly. The weight was measured using a digital balance (Torrey, model: LPCR-20/40, Querétaro, Mexico), the size of each animal was obtained by measuring the length from the base of the tail to the tip of the nose, and the abdomen diameter was estimated using the diaphragm zone as the upper limit and the fold of the legs as the bottom limit [16, 24]. The rats were placed in the ventral position to determine the abdominal circumference at the level of the largest abdomen using a nonextensible measuring tape (Rollfix, Hoechstmass, Germany) with an accuracy of 0.1 cm. The body mass index (BMI) was calculated using the formula weight/size^2^, and the fat percentage was calculated according to the Lee index for rodent models, with the formula: %fat = [(weight in g (0.33))/size in cm] × 100 [24].

### 2.4. Biochemical Assay

Commercial kits (Spinreact, Spain) and an automatic analyzer AutoKemII (KontroLab Company) were used to determine glucose, triglycerides, cholesterol, and low-density lipoprotein cholesterol (LDL-chol) and high-density lipoprotein cholesterol (HDL-chol) concentration in serum. The level of very-low-density lipoprotein (VLDL-chol) was obtained using the Friedenwald equation. Free fatty acid (FFA) concentration was determined according to the method described by Brunk and Swanson (1981). Plasma insulin concentrations were determined by an ELISA immunoassay (Diagnostica Internacional Company) with the resulting antibody-antigen complex assessed at 415 nm in a Stat fax 2600 plate reader (Wiener Lab Group). After a glucose load of 1.75 g/kg of body weight was administered orally (OGTT), blood was collected at 0, 30, 60, and 90 min for the quantitation of glucose and insulin, and the total area under curve (AUC) was calculated using the trapezoidal method, for both analytes [25, 26]. The HOMA-IR and HOMA-S indices were calculated according to the mathematical models used by Treviño et al. [16].

### 2.5. Intrahippocampal Injection of the A*β*_1–42_ Peptide

Once the MS model was induced, the animals treated with the respective diets (NCD and HCD) were subdivided into the four following experimental groups (*n* = 10/per group): (1) vehicle, (2) A*β*_1–42_, (3) MS, and (4) MS + A*β*_1–42_.

**Figure 1:**
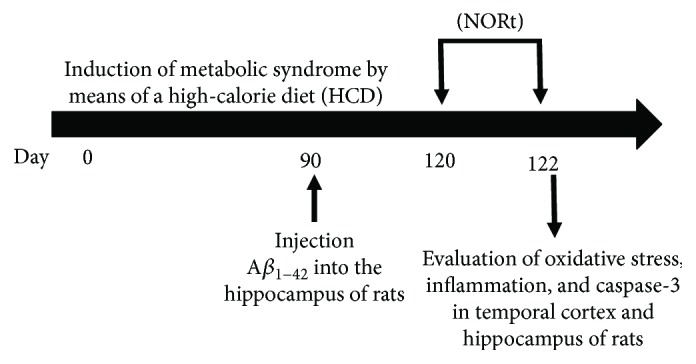
Timeline of feeding and behavioral and biochemical tests. Different assays made in the rats are shown. First, the rats were fed with a HCD; 90 days later, A*β*_1–42_ was injected into the hippocampus. Subsequently, we evaluated the NORt, and finally the rats were sacrificed for biochemical assays.

The peptide A*β*_1–42_ (Figure 1) was obtained from Sigma-Aldrich (St. Louis, MO, USA), which was dissolved in a physiology saline solution (SS) at a concentration of 5 mg/mL and incubated at 37°C for 72 h to induce aggregation. For surgical purposes, the animals were anesthetized with ketamine-xylazine (0.2 mL/100 g, i.p.) and placed onto a stereotaxic apparatus (Stoelting Co., Wood Dale, IL). Stereotaxic coordinates to produce a bilateral lesion into the hippocampus (Hp) (coordinates: A: −4.3 mm from the bregma, L: −3 mm from the midline, and V: −2.9 mm below the dura) followed Paxinos and Watson [27].

Injections of A*β*_1–42_ or vehicle (2 *μ*L) per side were administered for 10 min with a Hamilton syringe. After surgery, the animals were returned to their cages with free access to food (HCD or NCD) and water. They were administered antibiotic (Fluoroquinolone) daily for 5 days to recover from surgery.

### 2.6. Evaluation of the Novel Object Recognition Test (NORt)

Thirty days after the A*β*_1–42_ injection, the NORt was performed for the evaluation of recognition memory in rats. The NORt is based on the tendency of animals to spend more time exploring a novel object rather than a familiar one [23, 28, 29]. The rats were placed in a device with a square geometric shape (80 cm wide × 80 cm long × 80 cm high) to evaluate them in the open field. The first stage of the test is habituation, where the animals explored the box for 5 min. Subsequently, the rats returned to the domestic cage. In this phase, the distance traveled for each animal was quantified. Twenty-four hours later, the second stage, called familiarization, was performed where the animals were placed in the field with two similar objects for 5 min to quantify the inspection time for each object.

After two hours, the short-term recognition memory (STRM) was evaluated. In this test, one of the familiar objects was exchanged for a second new object in the same place in the box. The animal was placed back in the box to quantify the exploration time. The designated time for exploration of both the familiar and novel objects was 5 min for each object. The long-term memory recognition (LTMR) was evaluated 24 hours later. The same animals were exposed to two objects; one familiar object was exchanged by a third new object at the same location in the box, quantifying the exploration time of each object (familiar and novel) for 5 minutes.

STRM and LTRM was determined by the recognition index (RI) = (TN − TF)/(TN + TF) [23, 30], where TF and TN are the exploring times for each object, familiar and novel. To exclude odor cues, the open-field apparatus and the objects were cleaned with 80% ethanol after each session.

### 2.7. Redox Balance and Inflammation Measurement

Once the NORt was completed, the animals from each of the experimental groups were decapitated (*n* = 5 per group), their brains were removed and washed in an ice-cold SS, and the Hp and temporal cortex (TCx) were then dissected. The tissues were homogenized in 3 mL ice-cold 0.1 M phosphate buffered saline (PBS) (pH 7.4). The homogenate was centrifuged at 12,500 rpm at 4°C. The supernatant was obtained and stored at −70°C. The supernatants were used for the redox balance and inflammation assay.

#### 2.7.1. Reactive Oxygen Species Assay

We used 5 *μ*L of homogenized tissues, which were diluted in 9 volumes of 40 mM TRIS plus HEPES buffer and then incubated with 5 *μ*M 2′7′-dichlorodihydrofluorescein diacetate (DCFH-DA). The samples were incubated at 37°C for 1 h under constant shaking before the fluorescence signals were determined in a PerkinElmer LS50-B luminescence spectrometer at 488 nm excitation and 525 nm emission wavelengths. Values were obtained with a 2′7′-dichlorofluorescein (DCF) standard curve (Sigma-Aldrich). The results were expressed as nanomoles of DFC produced per milligram of protein per minute [16, 31, 32].

#### 2.7.2. Lipid Peroxidation Assay

The previously homogenized Hp and TCx tissues (1 mL) were added to 4 mL of a chloroform-methanol mixture (2 : 1, *v*/*v*). Samples were stirred and placed in ice for 30 min in the dark. The upper phase was discarded, and fluorescence of the chloroform phase was determined at 370 nm excitation and 430 nm emission wavelengths in a PerkinElmer LS50-B luminescence spectrometer. The sensitivity of the equipment was adjusted to a fluorescent signal of 140 fluorescence units (FU) with a standard quinine solution (0.001 mg/mL quinine in 0.05 M H_2_SO_4_). The results were expressed as relative fluorescence units (RFU) per milligram of protein [32].

#### 2.7.3. Assay to Quantify SOD and Catalase

Superoxide dismutase (SOD) was analyzed by the pyrogallol method. The pyrogallol in basic medium is self-oxidized generated in the superoxide radical reaction medium. In this way, the radical reaction is propagated, accelerating autooxidation by absorbing light at 420 nm. The presence of SOD inhibits autoxidation by avoiding propagation reactions. Tris-HCl buffer (0.05 M, pH 8.2; 2950 *μ*L) containing 1 mM Na_2_EDTA was placed in a quartz cuvette; then, 50 *μ*L of pyrogallol solution (12 mM in 1 mM HCl) and 50 *μ*L of homogenate (sample) were added to the buffer. Immediately after, the mixture was shaken vigorously and the reaction mixture was scanned every 30 seconds for 5 minutes, using a Lambda EZ-150 (PerkinElmer Company) at room temperature (23.8°C). By convention, 1 U of SOD is taken as the amount of enzyme that inhibits the pyrogallol autooxidation reaction by 50% at 25°C and pH 8.2.

Quantification of catalase activity (CAT) was performed using UV light spectrophotometry (Lambda EZ-150, PerkinElmer Company), as established by Nelson and Kiesow in 1972. The tissue samples were homogenized with 20 mM of potassium phosphate buffer (pH 7.4 and 150 mM NaCl, with 1 : 20 dilution) and centrifuged at 10,000 ×g/10 min/4°C. The experiment consisted of mixing 2.0 mL of potassium phosphate buffer (50 mM at pH 7.0), 0.05 mL of H_2_O_2_ (0.3 M), and 50 *μ*L of the homogenate. Changes in the absorption of H_2_O_2_ were followed for 60 seconds at 240 nm. Catalase activity was calculated as *μ*mol/min/mg protein, which is equal to U/mg protein.

#### 2.7.4. Measurement of IL-1*β* and TNF-*α*

The concentration of IL-1*β* and TNF-*α* in the homogenates of Hp and TCx was measured by the sandwich immunoassay procedure (R&D Systems, Minneapolis, MN) (Díaz et al. [19]). The intensity of color was in correlation with the amount of the cytokine. The lowest detection limits of these ELISA kits are 0.7 pg/mg and 1.0 pg/mg of protein for IL-1*β* and TNF-*α*, respectively.

### 2.8. Histological Examination

After the NORt, the rats of all experimental groups (*n* = 5/group) were anesthetized with sodium pentobarbital (40 mg/kg, i.p.) and perfused with 200 mL of 4% paraformaldehyde. The brains were removed and postfixed in the same fixative solution for 48 h and embedded in paraffin. 5 *μ*m thick coronal sections were taken from each brain at the level of the anterior temporal area, approximately 3.8 to 6.8 mm from the bregma. The coronal sections were placed on slides to be subsequently processed by immunohistochemical techniques.

#### 2.8.1. Evaluation of GFAP and Caspase-3 Reactivity

The paraffin was removed from the slides, and they were rehydrated. The nonspecific binding sites were blocked by incubating in 2% IgG-free bovine serum albumin (BSA, Sigma) at room temperature. Afterwards, the specimens were incubated with 0.2% Triton X-100 overnight at 4–8°C with primary antibodies: GFAP (1 : 1000, Millipore) to mark astrocytes and caspase-3 (casp-3; 1 : 100, Santa Cruz Biotechnology Inc.) that were determined by anti-rabbit FITC-labelled secondary antibodies (1 : 100, Jackson ImmunoResearch Laboratories Inc.). The slides were counterstained with Vectashield with DAPI (Vector Labs) for nuclear staining.

Photomicrographs were taken using a fluorescence microscope Olympus BX-41 and three consecutive slices of each brain tissue were used to observe CA1 hippocampal neurons at 40x. The number of GFAP- and casp-3-immunoreactive cells was quantified in the CA1 subfield of the Hp. Four fields per slide were analyzed and graphically expressed as an average per group ± SE. The images were taken using a digital camera acopled at BX-41 Olympus microscope and the analysis were did with Image Pro Premier of Media Cybernetics.

### 2.9. Statistical Analysis

The results were expressed as the mean ± standard error of the mean (SEM) for all experiments. The statistical zoometry data analysis, the biochemical parameters in serum, and HOMA indices were analyzed by a Wilcoxon signed-rank test, and the significance level was set at *p* ≤ 0.05. Meanwhile, NORt, ROS, lipid peroxidation, and antioxidant enzyme levels, as well as a number of GFAP- and casp-3-immunoreactive cells, were analyzed by a one-way ANOVA and followed by Bonferroni test, where *p* < 0.05 was considered significant.

## 3. Results

### 3.1. The High-Calorie Diet Induces Metabolic Syndrome

Rats administered with the HCD for three months showed an increase in the following zoometric parameters: body weight (18%, *p* = 0.0002), abdominal circumference (10%, *p* = 0.0002), BMI (34.5%, *p* < 0.0001), and Lee index (15%, *p* < 0.0001), which were significant in comparison to rats treated with NCD. On the other hand, the biochemical parameters showed the characteristics of the MS for the animals administered with HCD, showing a significant increase in FFA (34%, *p* < 0.0001), triglycerides (49%, *p* < 0.0001), and LDL-chol (23.6%, *p* = 0.0001) (Table 2) and a significant decrease in the HDL-chol fraction (47%, *p* = 0.0002), compared to the group fed with the control diet (NCD). Also, the metabolic alteration was observed in fasting and after an oral loading of glucose, in relation to the carbohydrate and its insulin response, with increases corresponding to 37.6% glucose AUC (*p* < 0.0001) and 42.8% insulin AUC (*p* < 0.0001) being observed. In the homeostasis model of insulin resistance assessment (HOMA-IR), insulin resistance was determined from an increase of 105% (*p* = 0.0003). Peripheral insulin sensitivity was assessed by an insulin sensitivity percentage (HOMA-S%) that decreased by 51.4% (*p* < 0.0001, Table 2). The results are in concordance with the reports of the MS parameters. Table 2 is reproduced from Treviño et al. [23].

### 3.2. Metabolic Syndrome and Injection of A*β*_1–42_ into the Hippocampus Impair Recognition Memory

**Figure 2:**
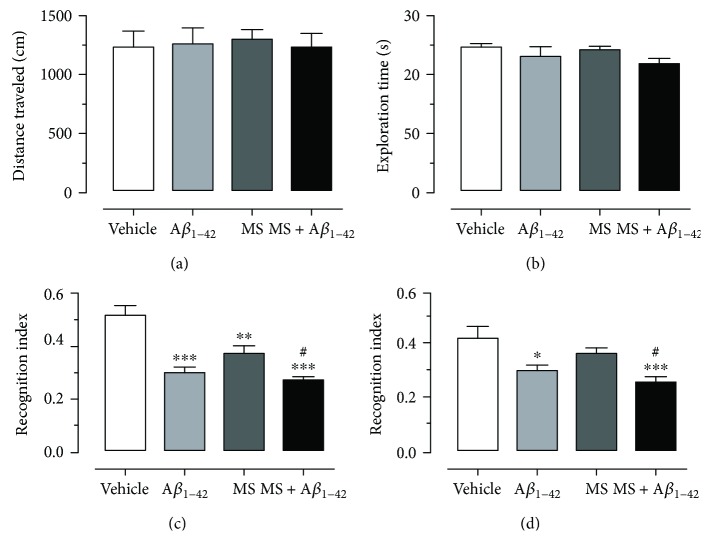
Metabolic syndrome and A*β*_1–42_ injection in the hippocampus decrease the recognition memory in the novel object recognition test (NORt) in rats. Habituation phase (motor activity) and exploration time are not different in the vehicle (*n* = 10), A*β*_1–42_ (*n* = 10), MS (*n* = 10), and MS+ A*β*_1–42_ (*n* = 10) groups (a, b). Several changes in the recognition index in the short-term recognition memory (STRM) and long-term memory (LTRM) can be observed in the MS and A*β*_1–42_ and their combination. The mean of each activity ± SE is plotted. Data were analyzed with one-way ANOVA and the posttest Bonferroni test (^∗^*p* < 0.05, ^∗∗^*p* < 0.01, and ^∗∗∗^*p* < 0.001 compared with the vehicle group). ^#^*p* < 0.05 compared with the MS group versus MS + A*β*_1–42_ groups.

Once the MS was generated in the animals, we proceeded to inject A*β*_1–42_ into the Hp and evaluate memory recognition. Thirty days after the injection, the NORt was evaluated. In the habituation phase, the locomotor activity of the animals of each experimental group was evaluated by means of the quantification of the distance covered in the box. The results indicated that the average distance traveled by the animals of the experimental groups was as follows: vehicle: 1246 ± 119, A*β*_1–42_: 1273 ± 130, MS: 1308 ± 82, and MS plus A*β*_1–42_: 1244 ± 96 (Figure 2(a)). Statistical analysis indicates that there is no significant difference in motor activity when comparing the A*β*_1–42_ group, the MS group, and the MS + A*β*_1–42_ group, with respect to the vehicle.

Twenty-four hours after the completion of the habituation phase, the NORt was performed. In this test, the STRM and LTRM were evaluated. At the beginning of the task, the exploration time that each animal performed, when exposed to the two similar objects (familiarization stage), was quantified. The results indicate that all the experimental groups recorded a latency time of exploration that oscillates between 22 sec and 25 sec; therefore, the statistical analysis does not indicate any significant differences between the experimental groups (Figure 2(b)).

Subsequently, to evaluate the STRM and LTRM, the exploration index was quantified with the formula previously described in the methodology section. The results indicate that when evaluating the STRM, the exploration index for the vehicle group, A*β*_1–42_ group, MS group, and SM + A*β*_1–42_ group was 0.52 ± 0.03, 0.31 ± 0.02, 0.37 ± 0.02, and 0.27 ± 0.01, respectively (Figure 2(c)). Meanwhile, the LTRM evaluation showed an exploration index of 0.43 ± 0.03, 0.30 ± 0.01, 0.36 ± 0.01, and 0.26 ± 0.01 for the vehicle group, A*β*_1–42_ group, MS group, and MS + A*β*_1–42_ group, respectively (Figure 2(d)).

These results indicate that the recognition index recorded from groups A*β*_1–42_, MS, and MS + A*β*_1–42_ was significantly lower in comparison to that recorded from the vehicle group, both in STRM and in LTRM (one-way ANOVA, *p* < 0.05). Likewise, it is observed that the MS + A*β*_1–42_ group presents a significant difference in the recognition index when compared to the A*β*_1–42_ group (11 and 17%) and MS group (27 and 30%) (one-way ANOVA, *p* < 0.05). This suggests that the MS exacerbates the deterioration of the recognition memory in rats with an injection of A*β*_1–42_ into the Hp.

### 3.3. Metabolic Syndrome and A*β*_1–42_ Induce Oxidative Stress in the Hippocampus and Temporal Cortex

**Figure 3:**
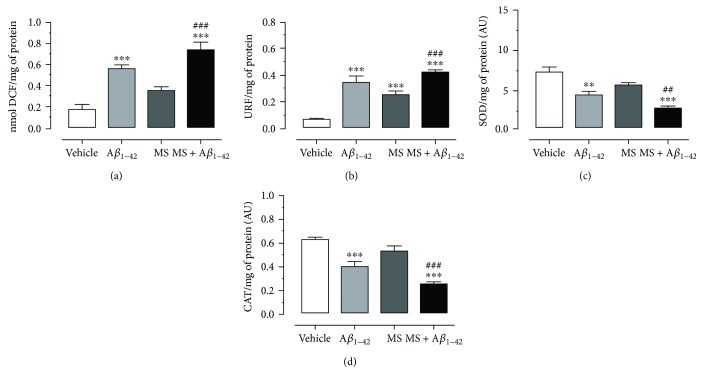
Effect A*β*_1–42_ on oxidative stress in the temporal cortex of rats with metabolic syndrome. (a) ROS assay. (b) Lipid peroxidation assay. (c) Superoxide activity assay. (d) Catalase activity assay. The mean of data ± SE is plotted. Data were analyzed with one-way ANOVA and the posttest Bonferroni test (^∗∗^*p* < 0.01 and ^∗∗∗^*p* < 0.001 compared with the vehicle group). ^##^*p* < 0.01 and ^###^*p* < 0.001 compared with the MS group versus MS + A*β*_1–42_ group.

**Figure 4:**
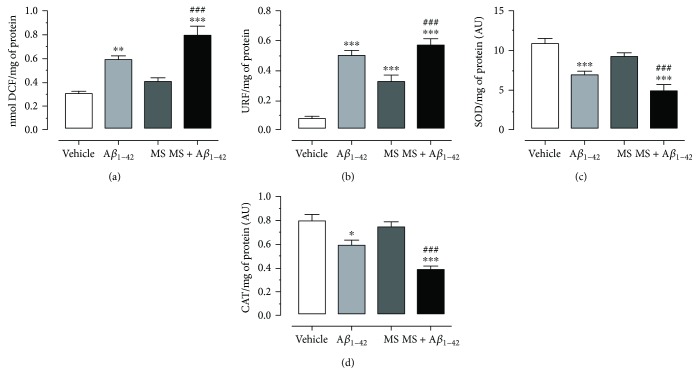
Effect A*β*_1–42_ on oxidative stress in the hippocampus of rats with metabolic syndrome. (a) ROS assay. (b) Lipid peroxidation assay. (c) Superoxide activity assay. (d) Catalase activity assay. The mean of data ± SE is plotted. Data were analyzed with one-way ANOVA and the posttest Bonferroni test (^∗^*p* < 0.05, ^∗∗^*p* < 0.01, and ^∗∗∗^*p* < 0.001 compared with the vehicle group). ^###^*p* < 0.001 compared with the MS group versus MS + A*β*_1–42_ group.

To evaluate oxidative stress, we proceeded to quantify the concentration of ROS, by means of the measurement of 2′,7′-dichlorodihydrofluorescein in the TCx and Hp of the experimental groups shown in Figures 3(a) and 4(a). The baseline values to TCx and Hp in the vehicle group were 0.18 ± 0.04 and 0.30 ± 0.02 nmol of DCF/mg protein/min, respectively. For the A*β*_1–42_ group and the MS group, the concentration obtained was 0.56 ± 0.03 and 0.60 ± 0.02 nmol of DCF/mg protein/min and 0.36 ± 0.03 and 0.42 ± 0.02 nmol of DCF/mg protein/min, respectively, while for the MS + A*β*_1–42_ group, the concentration recorded was 0.74 ± 0.07 and 0.80 ± 0.08 nmol of DCF/mg protein/min. These values indicate that the groups A*β*_1–42_, MS, and MS + A*β*_1–42_ present a significant increase in ROS production compared to vehicle group, in both the TCx and the Hp. Likewise, it is observed that the MS + A*β*_1–42_ group promotes an increased generation of ROS in these brain regions, compared to the A*β*_1–42_ group (30 and 32%), as well as to the MS (102 and 92%) group.

The lipid peroxidation measurement of the Hp and TCx is shown in Figures 3(b) and 4(b) (A*β*_1–42_ group (0.35 ± 0.04 and 0.48 ± 0.03 URF/mg protein), MS group (0.26 ± 0.02 and 0.31 ± 0.04 URF/mg protein), and MS + A*β*_1–42_ group (0.42 ± 0.01 and 0.55 ± 0.03 URF/mg protein)). All groups exhibited a significant increase in lipid peroxidation compared to the vehicle group, which presented a concentration of 0.07 ± 0.005 and 0.08 ± 0.01 URF/mg protein. Additionally, we observed that the A*β*_1–42_ + MS group had a higher level of lipid peroxidation, compared to the other groups, both in the TCx and in the Hp.

Figures 3(c), 3(d), 4(c), and 3(d) show the enzymatic activity SOD and CAT in the TCx and Hp. The TCx (Figure 3(c)) of the vehicle group presents 7.5 ± 0.6 SOD activity/mg of protein, while the A*β*_1–42_ and MS groups show a decrement in SOD activity (4.6 ± 0.4 and 5.8 ± 0.3 SOD activity/mg of protein). In the same way, the SOD activity in the MS + A*β*_1–42_ group decreases (2.8 ± 0.3 SOD activity/mg of protein). The CAT activity (Figure 3(d)) exhibits a similar behavior; the A*β*_1–42_ and MS groups show a decrement (0.45 ± 0.04 and 0.59 ± 0.04 CAT activity/mg of protein, resp.) in relation to the vehicle group (0.68 ± 0.01 CAT activity/mg of protein), and the combination of MS and A*β*_1–42_ exhibits a decrement in the same way (0.30 ± 0.01 CAT activity/mg of protein).

The SOD and CAT activity in the Hp is shown in Figure 4(c) and 4(d). The vehicle group has 10.9 ± 0.4 SOD activity/mg of protein and the A*β*_1–42_ and MS groups have 7.0 ± 0.4 and 9.3 ± 0.3 SOD activity/mg of protein, while the combined condition (MS + A*β*_1–42_) produced the major decrement in the enzymatic activity (5.1 ± 0.8 SOD activity/mg of protein). Finally, the Hp (Figure 4(d)) of the vehicle group exhibited 0.80 ± 0.05 CAT activity/mg of protein, while that of the A*β*_1–42_ and MS groups exhibited 0.59 ± 0.04 and 0.75 ± 0.03 CAT activity/mg of protein, respectively, and the MS + A*β*_1–42_ exacerbated this decrement (0.38 ± 0.03 CAT activity/mg of protein). Statistical analyses indicate a significant difference between the experimental groups and the vehicle group (^∗^*p* < 0.05, ^∗∗^*p* < 0.01, and ^∗∗∗^*p* < 0.001), as well as between the MS + A*β*_1–42_ group and the MS group (^###^*p* < 0.001).

### 3.4. The Metabolic Syndrome and A*β*_1–42_ Injection Increase the Concentration of IL-1*β* and TNF-*α* in the Temporal Cortex and Hippocampus

**Figure 5:**
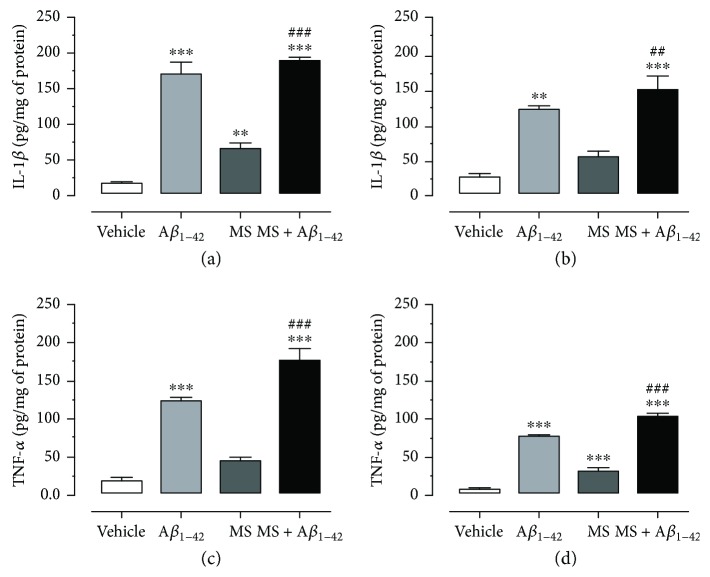
The injection of A*β*_1–42_ into the hippocampus increases the concentration of proinflammatory cytokines in HCD-fed rats. (a) IL-1*β* in the temporal cortex. (b) IL-1*β* in the hippocampus. (c) TNF-*α* in the temporal cortex. (d) TNF-*α* in the hippocampus. Metabolic syndrome induced by HCD and injection with A*β*_1–42_ increase the concentration of IL-1*β* and TNF-*α*, in the TCx and hippocampus (a–d). The mean of data ± SE is plotted. Data were analyzed with one-way ANOVA and the posttest Bonferroni test (^∗∗^*p* < 0.01 and ^∗∗∗^*p* < 0.001 compared with the vehicle group). ^###^*p* < 0.001 compared with the MS group versus MS + A*β*_1–42_ group.

The concentration of cytokines in the TCx (Figure 5(a)) and the Hp (Figure 5(b)) was obtained by ELISA. IL-1*β* levels in the TCx and Hp of the control rats were 17.9 ± 1.4 and 29.4 ± 3.1 pg/mg of protein, respectively, while for MS induced by the HCD group, the IL-1*β* concentration was 68.6 ± 3.3 pg/mg of protein in the TCx and 59.05 ± 4.6 pg/mg of protein in the Hp. The A*β*_1–42_ group showed a concentration of 172.6 ± 14.3 pg/mg of protein in the TCx and 125.6 ± 2.5 pg/mg of protein in the Hp. Likewise, the IL-1*β* concentration in the MS + A*β*_1–42_ group increased in the TCx (191.3 ± 2.2 pg/mg of protein) and in the Hp (152.5 ± 19.7 pg/mg of protein).

The level of TNF-*α* in the TCx and Hp is shown in Figures 5(c) and 5(d), respectively. We found a concentration of 19.45 ± 2.8 (TCx) and 5.4 ± 0.5 pg/mg of protein (Hp) in the control group. In the A*β*_1–42_ group, high concentrations (124.9 ± 3.2 pg/mg of protein in the TCx and 74.2 ± 3.1 pg/mg of protein in the Hp) were observed. The concentration was also incremented in the MS group, both in the TCx and in the Hp (45.8 ± 3.5 and 31.07 ± 1.2 pg/mg of protein, resp.). For the combined condition MS + A*β*_1–42_, the concentration in the TCx and Hp of TNF-*α* was the highest: 176.3 ± 14.9 and 100.9 ± 2.3 pg/mg of protein, respectively. Statistical analyses indicate that there exists a significative difference between the groups (one-way ANOVA, Bonferroni's test; ^∗∗^*p* < 0.01 and ^∗∗∗^*p* < 0.001 in relation to the vehicle group and ^###^*p* < 0.001 in relation to the MS group versus the MS + A*β*_1–42_ group).

### 3.5. The Injection of A*β*_1–42_ and Metabolic Syndrome Increased GFAP Immunoreactivity in the Temporal Cortex and Hippocampus

**Figure 6:**
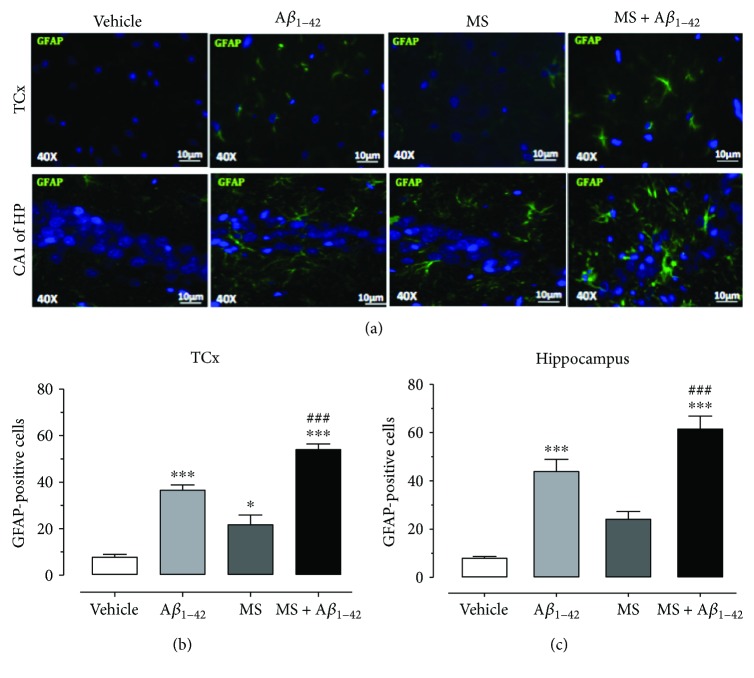
Effect of the injection of A*β*_1–42_ into the hippocampus of rats with HCD-induced MS on neuronal inflammation. A*β*_1–42_ in the hippocampus of HCD-fed rats increases the number of GFAP-positive cells in the TCx and the hippocampus (a–c). The mean of data ± SE is plotted. Data were analyzed with one-way ANOVA and the posttest Bonferroni test (^∗^*p* < 0.05, ^∗∗^*p* < 0.01, and ^∗∗∗^*p* < 0.001 compared with the vehicle group). ^##^*p* < 0.01 compared with the MS group.

As a first approach in the understanding of the inflammatory response that an HCD causes in rat's brain, we studied the immunoreactivity of GFAP in the TCx and CA1 subfield of the Hp, as an indicator of the activation of astrocytes after the chronic administration of an HCD and an A*β*_1–42_ injection into the Hp. Figure 5(a) shows that the immunoreactivity of GFAP (green color) from HCD-treated rats was increased and distributed widely in the region of the TCx and CA1 subfield of the Hp, as the photomicrographs reveal when compared to the immunoreactivity of GFAP from the control group. The quantification of the GFAP-positive cells in the TCx was 8 ± 1 cells in the vehicle group, in contrast with the A*β*_1–42_ and MS groups, where the value was 36 ± 2 and 22 ± 2 cells, respectively (Figure 6(b)), whereas the MS + A*β*_1–42_ presented 54 ± 2 cells positive for GFAP. An increment in GFAP was also observed in the Hp, since the control group presented 8 ± 1 cells, while the A*β*_1–42_ and MS groups presented 44 ± 5 and 24 ± 3 cells, respectively. Finally, the MS + A*β*_1–42_ group presented the major increment in GFAP-positive cells (62 ± 5 cells, Figure 5(c)). One-way ANOVA analysis with Bonferroni's test indicates a significant difference of ^∗^*p* < 0.05, ^∗∗^*p* < 0.01, and ^∗∗∗^*p* < 0.001 in relation to the vehicle group and ^##^*p* < 0.001 between MS and MS + A*β*_1–42_ groups.

### 3.6. Caspase-3 Is Incremented in the Temporal Cortex and Hippocampus of Rats with Metabolic Syndrome and A*β*_1–42_ Injection

**Figure 7:**
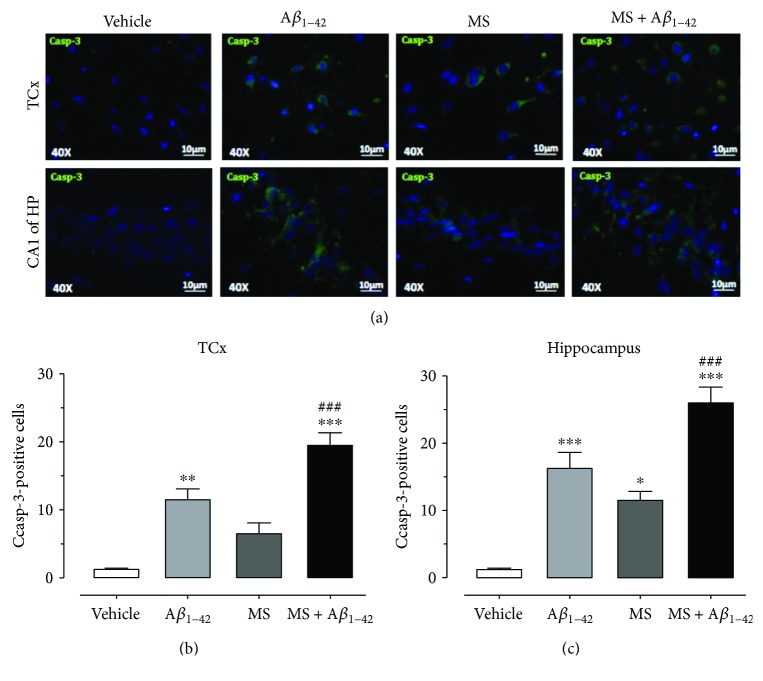
Effect of the injection of A*β*_1–42_ into the hippocampus of rats with HCD-induced MS on neuronal death. A*β*_1–42_ in the hippocampus of HCD-fed rats increases the number of caspase-3-positive cells in the TCx and the hippocampus (a–c). The mean of data ± SEM is plotted. Data were analyzed with one-way ANOVA and the posttest Bonferroni test (^∗^*p* < 0.05, ^∗∗^*p* < 0.01, and ^∗∗∗^*p* < 0.001 compared with the vehicle group). ^###^*p* < 0.001 compared with the MS group.

We measured the neuronal death induced by OS and inflammatory processes with casp-3-positive cells in the TCx and Hp of the rats fed with HCD and injected with A*β*_1–42_.  Figure 7(a) shows the mark for casp-3 (green color), which is widely distributed in both nuclei. The quantification of casp-3-positive cells is shown in Figure 7(b) for the TCx and Figure 7(c) for the Hp. The vehicle group presented 1 ± 0.25 casp-3-positive cell in both the TCx and Hp, while the A*β*_1–42_ group exhibited 11 ± 1 and 16 ± 2 positive cells in the TCx and Hp, respectively. Analysis of the MS group also showed an increase in casp-3-positive cells in the TCx (6 ± 1 cells) and Hp (11 ± 1 cells). However, the highest count was registered in the SM + A*β*_1–42_ group, in both the TCx (19.5 ± 2 cells) and Hp (26 ± 2 cells). Statistical analyses indicate a significant difference between the groups (^∗^*p* < 0.05, ^∗∗^*p* < 0.01, and ^∗∗∗^*p* < 0.001) in relation to the vehicle group, as well as between the SM and SM + A*β*_1–42_ groups (^###^*p* < 0.001).

## 4. Discussion

Data obtained in this work show that the injection of A*β*_1–42_ into the Hp of rats with MS produces a major impairment in the recognition memory, as demonstrated by the NORt. This event could be explained by the exacerbated increment in ROS and the decrement in the antioxidant system in the Hp and TCx, associated with inflammation and death processes. Our results indicate that MS plays a key role, since it aggravates the process of dementia pathologies, such as AD.

MS was induced in rats according to Treviño et al. [16]. Briefly, the rats were fed with a HCD for 90 days, and we found elevated compatible biomarkers, such as insulin resistance by hyperglycemia, and elevated levels of VLDL-chol and LDL-chol, free fatty acids, and triglycerides, as well as a decrement in HDL-chol (Table 2) in humans with a diagnosis of MS. Also, it is important to mention that BMI, abdominal circumference, and adiposity were also increased [23].

It has been shown that a diet plays an important role in the appearance of MS in rats [23, 28, 33]. In this work, we show that MS induced by a HCD can produce a memory decrement in the NORt (Figure 1). The data are in concordance with those of different studies that have demonstrated a significant decrement in cognitive processes in animals with MS, evaluated in the water maze [16, 28], the NORt [23], and the Y-maze [33]. This memory decrement is explained by a deterioration of brain areas related to cognitive processes like the Hp when there exists a synaptic loss [34]. In this sense, Treviño et al. in 2017 demonstrated that chronic feeding with a HCD produces a significant shrinkage of the length of dendritic arborization and a loss of synaptophysin in the Golgi-stained Hp [23]. These results indicate that MS may be involved in the onset and development of dementia illnesses, such as AD, which is characterized by the presence of senile plaques containing A*β* deposits and neurofibrillary tangles composed of the tau protein in a hyperphosphorylated state [35]. Specifically, A*β* deposits are relevant because it has been shown that a high-fat diet induces the appearance of A*β* deposits in the dental gyrus of the Hp of C57BL/6J mice [5].

The link between the consumption of a HCD and the development of AD is poorly understood. However, in order to determine the mechanisms involved in this process, A*β*_1–42_ was injected into the Hp of rats, with and without MS induced by a HCD, producing an exacerbated decrement in the recognition index in the NORt (Figure 2). Our results are in accordance with those of Park et al. because they demonstrate that the injection of A*β*_25–35_ into diabetic rats produces an increment in the latency to locate the platform in the water maze test [36]. However, MS characterized by insulin resistance changes in glucose uptake and dyslipidemia with over fluxes of triglycerides and FFA is an early stage of metabolic disorders such as diabetes, and the impairment of cognition could be influenced by an imbalance in the management of these macromolecules in the TCx and Hp. Principally, this cognitive event could be explained by the insulin-resistant state observed in MS and diabetes [37]. Insulin participates in neuromodulation: regulating neurotransmitter concentrations such as acetylcholine [38], a mediator of long-term potentiation and learning and memory processes [39]. Likewise, insulin can modulate intracellular accumulation of A*β*, due to insulin pathways that stimulate the amyloid extracellular secretion in the brain [13, 40, 41]. This event is mediated by insulin, as well as by the insulin-like growth factor through mediated Akt phosphorylation/inactivation of glycogen synthase kinase-3*β* (Akt/GSK-3*β*), and defects in the signaling lead to hyperphosphorylation of the tau protein [42].

On the other hand, in both the TCx and Hp, the MS and injection of A*β*_1–42_ separately trigger a high ROS and lipid peroxidation production, which was confirmed in our results. Additionally, we also show that injection of A*β*_1–42_ into the Hp of rats with MS enhanced the ROS and lipid peroxidation production in an additive manner, in both brain zones (Figures 3(a), 3(b), 4(a), and 4(b)). In physiological conditions, there is a delicate balance between the synthesis of ROS and the activities of antioxidant pathways. In both AD and MS, OS is induced; prooxidant pathways are activated in dyslipidemia and hyperglycemia states associated with nonenzymatic and Millard reactions and increase the advanced glycation end-products (AGEs) and hydroxyl radicals, favoring the high level of the superoxide anion and, consequently, products such as 4-hydroxynonenal and malondialdehyde [43]. Likewise, there is evidence indicating that A*β* induces OS within the lipid bilayer, in which A*β*_1–42_ inserts as oligomers and serves as a source of ROS, as well as a lipid peroxidation initiator. OS, lipid peroxidation, and increased production of ROS reduce the activity of proteins that are critical for neuronal homeostasis. The consequence of this event is the activity decrement in CAT and SOD, the main antioxidant enzymes in neurons, as shown in both nuclei (Figures 3(c), 3(d), 4(c), and 4(d)). These results are in agreement with those of numerous studies that demonstrate that patients with MS have a high risk of developing AD, due to high levels of oxidative metabolism that affects cell structure, leading to neuronal damage [44] contributing to the neurodegenerative process and explaining the loss of the cognitive process.

Inflammation is another key factor in the development of AD [13]. The inflammatory process exists in the production of chemokine, ROS, and proinflammatory cytokines that carry a dysregulation in the immune response and due to neurodegeneration [35]. Previous studies have suggested that the injection of A*β*_25–35_ into the TCx produces an increment in the expression of inducible nitric oxide synthase (iNOS), which induces a major NO synthesis and accelerates the formation of interleukin-1*β* (IL-1*β*) and tumor necrosis factor-*α* (TNF-*α*), both of which are involved in proinflammatory processes [19, 45]. In the same way, impaired insulin signaling and inflammation appear to be shared processes in T2DM and AD [5, 6, 20–22, 46], as well as in MS as suggested by our results. In T2DM, the c-Jun N-terminal kinase (JNK) pathway is stimulated by the TNF-*α* cascade, thereby initiating peripheral insulin resistance, causing IRS-1 inhibition at the brain level and the interruption of downstream cascade favoring tau hyperphosphorylation and amyloid assembly. IL-6 and other proinflammatory cytokines in MS maintain a low-grade chronic inflammation (inflammaging), which sustain inflammatory and upregulated pathways in the brain and a high probability of neurodegeneration. Figures 5(a)–5(d) show that the injection of the peptide A*β*_1–42_ into the Hp produces an increment in IL-1*β* and TNF-*α*. Similarly, these markers also increase in MS and are exacerbated with the combination of MS + A*β*_1–42_. In addition, it is known that in MS, the adipose tissue produces different cytokines [47], such as IL-1*β* and TNF-*α*, that are related to low levels of adiponectin [9], the development of MS and T2DM [48], and the appearance of AD [13, 49]. The peripheral inflammation is associated with increments in the GFAP immunofluorescence in astrocytes of the Hp and TCx, confirming the existence of inflammation in the brain of HD-induced MS rats. The immunoreactivity is greater in the MS + A*β*_1–42_ group. These results are in accord with those of studies that found that a high-fat diet produces an increment in microglial activation in the Hp of 3XTgAD mice [33] and GFAP in the Hp of C57BL/6 mice [5].

The brain damage induced by OS, neuroinflammation, and metabolic dysregulation can be measured by casp-3, a protein involved in the final stages of neuronal death by the apoptotic pathway [46]. Treviño et al. showed that a HCD can produce an increment in casp-3 [16]. In the same way, several studies have demonstrated that casp-3 participates in neuronal death induced by A*β* peptides [19, 50]. In this sense, we observed a high immunoreactivity of casp-3 in the Hp and the TCx of the MS and A*β*_1–42_ groups. However, when we combined both conditions (MS + A*β*_1–42_), neuronal death was incremented in such a way that suggests that MS can accelerate neuronal damage induced by A*β*_1–42_, and this neuronal death is responsible for the decrement in the NORt.

## 5. Conclusions

Finally, with our results, we can conclude that MS induced by a HCD in rats is a key factor in the neurodegeneration in the Hp and TCx, important nuclei in the generation and consolidation of memory, due to increases in the oxidative and inflammatory process provoked by the metabolic disorder. However, when A*β* is present in these brain zones, they aggravate the neurodegenerative process and even cognitive problems are accentuated. Therefore, we propose that MS is a determinant for the appearance of neurodegenerative diseases like AD.

## Figures and Tables

**Table 1:** Caloric composition of diets.

Composition	Caloric percentage (kcal/g) (LabDiet 5010)	Caloric percentage (kcal/g) (HCD (MX/E/2013/047377))
Carbohydrates	56.36	77.82
Proteins	13.02	7.95
Fat	27.66	14.22
Fiber (crude)	2.95	0.0
Ash	0.0	0.0
Total	99.99	99.99

**Table 2:** Zoometric and metabolic parameters to define metabolic syndrome.

	NCD	HCD	*p* value
	*n* = 20	*n* = 20	
*Zoometric panel*			
Weight (g)	337 ± 2.9	397 ± 4.8^∗∗∗^	0.0002
Abdominal perimeter (cm)	17.1 ± 0.2	18.8 ± 0.04^∗∗∗^	0.0002
Body mass index	0.9 ± 0.01	1.21 ± 0.02^∗∗∗^	<0.0001
Lee index	34.3 ± 0.1	39.5 ± 0.21^∗∗∗^	<0.0001
*Lipid panel*			
FFA (mg/dL)	2.21 ± 0.04	5.17 ± 0.11^∗∗∗^	<0.0001
Triglycerides (mg/dL)	71.1 ± 1.9	105.8 ± 2.6^∗∗∗^	<0.0001
Total cholesterol (mg/dL)	102.8 ± 9.1	99.5 ± 9.3	0.8666
*Cholesterol subfractions*			
VLDL-chol (mg/dL)	14.4 ± 1.2	21.2 ± 2.9	0.9108
LDL-chol (mg/dL)	20.3 ± 4	45.4 ± 3.5^∗∗∗^	0.0001
HDL-chol (mg/dL)	68.1 ± 2	32.9 ± 5.5^∗∗∗^	0.0002
*OGTT (1.75* g*/kg)*			
Glucose fasting (mg/dL)	90 ± 4.3	108 ± 4.3^∗∗^	0.009
Glucose 30 min (mg/dL)^‡^	129.5 ± 3.3	168 ± 8.4^∗∗^	0.0012
Glucose 60 min (mg/dL)^‡^	100.4 ± 2.9	155.7 ± 6.6^∗∗∗^	0.0002
Glucose 90 min (mg/dL)^‡^	91.5 ± 4.0	123.9 ± 5.4^∗∗^	0.0014
AUC glucose (mg/dL/90 min)	320.7 ± 3.6	439 ± 6.2^∗∗∗^	<0.0001
*Insulin curve*			
Insulin fasting (*μ*UI/mL)	9.0 ± 1.1	18 ± 3.2^∗∗^	0.0054
Insulin 30 min (*μ*UI/mL)^‡^	21.2 ± 2.8	56 ± 3.1^∗∗∗^	0.0002
Insulin 60 min (*μ*UI/mL)^‡^	13.9 ± 1.4	32.3 ± 1.8^∗∗∗^	0.0001
Insulin 90 min (*μ*UI/mL)^‡^	11.5 ± 1.0	25.8 ± 2.2^∗∗∗^	0.0003
AUC insulin (*μ*UI/mL/90 min)	45.4 ± 1.8	110.2 ± 2.7^∗∗∗^	<0.0001
HOMA-IR	1.17 ± 0.05	2.4 ± 0.3^∗∗∗^	0.0003
HOMA-S%	85.8 ± 3.2	41.7 ± 1.7^∗∗∗^	<0.0001

The results of zoometric and serum parameters are represented as the average of 20 separate experiments ± SEM. ∗∗ and ∗∗∗ indicate a significant difference between HCD and NCD groups with *p* < 0.05 by the nonparametric Wilcoxon test. ^‡^Values obtained after an oral glucose tolerance test (OGTT, 1.75 g of glucose/kg body weight). FFA: free fatty acid; VLDL: very-low-density lipoprotein; LDL: low-density lipoprotein; HDL: high-density lipoprotein; AUC: area under curve; HOMA-IR: homeostasis model assessment of insulin resistance; HOMA-S%: homeostasis model assessment of insulin sensitivity percentage. Table 2 is reproduced from Treviño et al. [23].

## References

[1] McGill A.-T. (2014). Causes of metabolic syndrome and obesity-related co-morbidities part 1: a composite unifying theory review of human-specific co-adaptations to brain energy consumption. *Archives of Public Health*.

[2] Freeman L. R., Haley-Zitlin V., Rosenberger D. S., Granholm A.-C. (2013). Damaging effects of a high-fat diet to the brain and cognition: a review of proposed mechanisms. *Nutritional Neuroscience*.

[3] Kassi E., Pervanidou P., Kaltsas G., Chrousos G. (2011). Metabolic syndrome: definitions and controversies. *BMC Medicine*.

[4] De Felice F. G., Lourenco M. V. (2015). Brain metabolic stress and neuroinflammation at the basis of cognitive impairment in Alzheimer’s disease. *Frontiers in Aging Neuroscience*.

[5] Busquets O., Ettcheto M., Pallàs M. (2017). Long-term exposition to a high fat diet favors the appearance of *β*-amyloid depositions in the brain of C57BL/6J mice. A potential model of sporadic Alzheimer’s disease. *Mechanisms of Ageing and Development*.

[6] Vykoukal D., Davies M. G. (2011). Vascular biology of metabolic syndrome. *Journal of Vascular Surgery*.

[7] Tsigos C., Kyrou I., Chala E. (1999). Circulating tumor necrosis factor alpha concentrations are higher in abdominal versus peripheral obesity. *Metabolism - Clinical and Experimental*.

[8] Hotamisligil G. S., Arner P., Caro J. F., Atkinson R. L., Spiegelman B. M. (1995). Increased adipose tissue expression of tumor necrosis factor-alpha in human obesity and insulin resistance. *The Journal of Clinical Investigation*.

[9] Wang B., Trayhurn P. (2006). Acute and prolonged effects of TNF-*α* on the expression and secretion of inflammation-related adipokines by human adipocytes differentiated in culture. *Pflügers Archiv*.

[10] Lionetti L., Mollica M. P., Lombardi A., Cavaliere G., Gifuni G., Barletta A. (2009). From chronic over nutrition to insulin resistance: the role of fat-storing capacity and inflammation. *Nutrition, Metabolism and Cardiovascular Diseases*.

[11] Argente-Arizón P., Freire-Regatillo A., Argente J., Chowen J. A. (2015). Role of non-neuronal cells in body weight and appetite control. *Frontiers in Endocrinology*.

[12] Varnum M. M., Ikezu T. (2012). The classification of microglial activation phenotypes on neurodegeneration and regeneration in Alzheimer’s disease brain. *Archivum Immunologiae et Therapiae Experimentalis*.

[13] Rojas-Gutierrez E., Muñoz-Arenas G., Treviño S. (2017). Alzheimer’s disease and metabolic syndrome: a link from oxidative stress and inflammation to neurodegeneration. *Synapse*.

[14] Moneim A. (2015). Oxidant/antioxidant imbalance and the risk of Alzheimer’s disease. *Current Alzheimer Research*.

[15] Chou C. L., Lin H., Chen J. S., Fang T. C. (2017). Renin inhibition improves metabolic syndrome, and reduces angiotensin II levels and oxidative stress in visceral fat tissues in fructose-fed rats. *PLoS One*.

[16] Treviño S., Aguilar-Alonso P., Flores Hernandez J. A. (2015). A high calorie diet causes memory loss, metabolic syndrome and oxidative stress into hippocampus and temporal cortex of rats. *Synapse*.

[17] Cass S. P. (2017). Alzheimer’s disease and exercise: a literature review. *Current Sports Medicine Reports*.

[18] Chiaravalloti A., Fiorentini A., Francesco U. (2016). Is cerebral glucose metabolism related to blood-brain barrier dysfunction and intrathecal IgG synthesis in Alzheimer disease?: a 18F-FDG PET/CT study. *Medicine*.

[19] Díaz A., Rojas K., Espinosa B. (2014). Aminoguanidine treatment ameliorates inflammatory responses and memory impairment induced by amyloid-beta 25-35 injection in rats. *Neuropeptides*.

[20] Solfrizzi V., Scafato E., Capurso C. (2011). Metabolic syndrome, mild cognitive impairment, and progression to dementia: the Italian Longitudinal Study on Aging. *Neurobiology of Aging*.

[21] Haan M. N. (2006). Therapy insight: type 2 diabetes mellitus and the risk of late-onset Alzheimer’s disease. *Nature Clinical Practice Neurology*.

[22] Nuzzo D., Picone P., Baldassano S. (2015). Insulin resistance as common molecular denominator linking obesity to Alzheimer’s disease. *Current Alzheimer Research*.

[23] Treviño S., Vázquez-Roque R. A., López-López G. (2017). Metabolic syndrome causes recognition impairments and reduced hippocampal neuronal plasticity in rats. *Journal of Chemical Neuroanatomy*.

[24] Rogers P., Webb G. P. (1980). Estimation of body fat in normal and obese mice. *The British Journal of Nutrition*.

[25] Potteiger J. A., Jacobsen D. J., Donnelly J. E. (2002). A comparison of methods for analyzing glucose and insulin areas under the curve following nine months of exercise in overweight adults. *International Journal of Obesity and Related Metabolic Disorders*.

[26] Purves R. D. (1992). Bias and variance of extrapolated tails for area-under-the-curve (AUC) and area-under-the-moment-curve (AUMC). *Journal of Pharmacokinetics and Biopharmaceutics*.

[27] Paxinos G., Watson C. (1998). *The Rat Brain in Stereotaxic Coordinates*.

[28] Lemos C., Rial D., Gonçalves F. Q. (2016). High sucrose consumption induces memory impairment in rats associated with electrophysiological modifications but not with metabolic changes in the hippocampus. *Neuroscience*.

[29] Vidal B., Vazquez-Roque R. A., Gnecco D. (2017). Curcuma treatment prevents cognitive deficit and alteration of neuronal morphology in the limbic system of aging rats. *Synapse*.

[30] Antunes M., Biala G. (2012). The novel object recognition memory: neurobiology, test procedure, and its modifications. *Cognitive Processing*.

[31] Ali S. F., David S. N., Newport G. D. (1993). Age-related susceptibility to MPTP-induced neurotoxicity in mice. *Neurotoxicology*.

[32] Pérez-Severiano F., Santamaría A., Pedraza-Chaverri J., Medina-Campos O. N., Ríos C., Segovia J. (2004). Increased formation of reactive oxygen species, but no changes in glutathione peroxidase activity, in striata of mice transgenic for the Huntington’s disease mutation. *Neurochemical Research*.

[33] Knight E. M., Martins I. V. A., Gümüsgöz S., Allan S. M., Lawrence C. B. (2014). High-fat diet-induced memory impairment in triple-transgenic Alzheimer’s disease (3xTgAD) mice is independent of changes in amyloid and tau pathology. *Neurobiology of Aging*.

[34] Grillo C. A., Piroli G. G., Lawrence R. C. (2015). Hippocampal insulin resistance impairs spatial learning and synaptic plasticity. *Diabetes*.

[35] Minter M. R., Taylor J. M., Crack P. J. (2016). The contribution of neuroinflammation to amyloid toxicity in Alzheimer’s disease. *Journal of Neurochemistry*.

[36] Park S., Kim D. S., Kang S., Moon N. R. (2013). *β*-Amyloid-induced cognitive dysfunction impairs glucose homeostasis by increasing insulin resistance and decreasing *β*-cell mass in non-diabetic and diabetic rats. *Metabolism - Clinical and Experimental*.

[37] Correia S. C., Santos R. X., Perry G., Zhu X., Moreira P. I., Smith M. A. (2011). Insulin-resistant brain state: the culprit in sporadic Alzheimer’s disease?. *Ageing Research Reviews*.

[38] de la Monte S. M., Chen G. J., Rivera E., Wands J. R. (2003). Neuronal thread protein regulation and interaction with microtubule-associated proteins in SH-Sy5y neuronal cells. *Cellular and Molecular Life Sciences CMLS*.

[39] Plum L., Schubert M., Brüning J. C. (2005). The role of insulin receptor signaling in the brain. *Trends in Endocrinology and Metabolism*.

[40] Moloney A. M., Griffin R. J., Timmons S., O’Connor R., Ravid R., O’Neill C. (2010). Defects in IGF-1 receptor, insulin receptor and IRS-1/2 in Alzheimer’s disease indicate possible resistance to IGF-1 and insulin signalling. *Neurobiology of Aging*.

[41] Wada A., Yokoo H., Yanagita T., Kobayashi H. (2005). New twist on neuronal insulin receptor signaling in health, disease, and therapeutics. *Journal of Pharmacological Sciences*.

[42] Phiel C. J., Wilson C. A., Lee V. M. Y., Klein P. S. (2003). GSK-3*α* regulates production of Alzheimer’s disease amyloid-*β* peptides. *Nature*.

[43] Luque-Contreras D., Carvajal K., Toral-Rios D., Franco-Bocanegra D., Campos-Peña V. (2014). Oxidative stress and metabolic syndrome: cause or consequence of Alzheimer’s disease?. *Oxidative Medicine and Cellular Longevity*.

[44] Baker L. D., Cross D. J., Minoshima S., Belongia D., Watson G. S., Craft S. (2011). Insulin resistance and Alzheimer-like reductions in regional cerebral glucose metabolism for cognitively normal adults with prediabetes or early type 2 diabetes. *Archives of Neurology*.

[45] Diaz A., Limon D., Chavez R., Zenteno E., Guevara J. (2012). A*β*_25–35_ injection into the temporal cortex induces chronic inflammation that contributes to neurodegeneration and spatial memory impairment in rats. *Journal of Alzheimer's Disease*.

[46] Stuart Wood I., de Heredia F. P., Wang B., Trayhurn P. (2009). Cellular hypoxia and adipose tissue dysfunction in obesity: symposium on ‘Frontiers in adipose tissue biology’. *The Proceedings of the Nutrition Society*.

[47] Højlund K., Frystyk J., Levin K., Flyvbjerg A., Wojtaszewski J. F. P., Beck-Nielsen H. (2006). Reduced plasma adiponectin concentrations may contribute to impaired insulin activation of glycogen synthase in skeletal muscle of patients with type 2 diabetes. *Diabetologia*.

[48] Kang S., Lee Y. H., Lee J. E. (2017). Metabolism-centric overview of the pathogenesis of Alzheimer’s disease. *Yonsei Medical Journal*.

[49] Dingman A., Lee S. Y., Derugin N., Wendland M. F., Vexler Z. S. (2006). Aminoguanidine inhibits caspase-3 and calpain activation without affecting microglial activation following neonatal transient cerebral ischemia. *Journal of Neurochemistry*.

[50] Hwang S., Lim J., Kim H. (2017). Inhibitory effect of lycopene on amyloid-*β*-induced apoptosis in neuronal cells. *Nutrients*.

